# Practical Notes and Formulæ

**Published:** 1883-11-20

**Authors:** 


### PRACTICAL NOTES AND FORMULÆ.

**Treatment of Catarrh.**—The following combination is stated by M. P. Vigier to have been used with good success in catarrhal affections:

| | | | | |
|---|---|---|---|---|
| ℞ Picis liquidæ | 1.0 gm.=grs. | 15, |
| Benzoini | 1.0 " | " 15, |
| Pulv. ipecac et opii | 1.0 " | " 15. |

Mix. Make into ten pills. Take three pills daily between meals.—*New Remedies.*

**Iron Rust Spot Remover.**—

| | |
|---|---|
| Cream of tartar | 50 parts, |
| Binoxalate of potassium, powdered | 50 parts, |
| Oil of rosemary | 1 part. |

Rub to powder and mix well. Moisten the spot, place on a heated tin plate and rub with the moistened powder.—*New Idea.*

**"Anti-Spree Mixture.**—

| | |
|---|---|
| Pepsin, pure (in scales) | 20 parts, |
| Water | 2,000 " |
| Hydrochloric acid | 15 " |
| Oil of sassafras | 6 " |

Mix. Shake well. Take a tablespoonful every hour.

Half-ounce doses of *Liq. ammon. acetatis* at intervals of two to four hours in water is a favorite in many hospitals, and three drops of tincture of nux vomica, in a little water, taken every hour, is a good remedy for bracing up on. These mixtures are chiefly serviceable for quieting the disturbed stomach, but are of little value in relieving the disorganization of the nervous system that ends in delirium.—*New Remedies.*

**To Protect Surgical Instruments.**—The following is recom mended by Prof. Olmstead, of Yale College, (Pop. Sci. News): Melt slowly together six or eight parts of lard to one of resin, and stir until it is cool. Rubbed on a bright metalic surface, it protects the polish effectually. It can be wiped off nearly clean, if it is desired, as in case of knife blades, or it can be thinned with coal oil or benzine. The surface should be both bright and dry, as it will not prevent the continuance of oxidation already begun.—*Med. and Surg. Reporter.*

**Oxide of Zinc in Diarrhœa.**—M. Gubler has found it most useful in the diarrhœa of phthisis, and whenever ulceration of the uterus is suspected. He gives it in powders in the form:

| | |
|---|---|
| ℞ Oxide of zinc | grs. xxx, |
| Bi-carbonate of soda | grs. x. |

In four powders, two or three daily.

**Follicular Pharyngitis.**—Dr. Shoemaker, of Kansas, in Peoria Medical Monthly, says: "For this affection, for internal topical applications I find the following to prove satisfactory when used as directed:

℞ Tannic acid ........................ ℥ ij,

Iodoform ........................ grs. xx.

M. And use upon the parts by means of the insufflator, and care must be taken in selecting and using the instrument in this disease. A glycerite of tannic acid and iodoform, as the following, is excellent:

℞ Tannic acid ........................ ʒ ij,

Iodoform ........................ grs. cxx,

Glycerine pura ........................ pt. ½.

M. And apply to the throat with camel's hair brush, according to circumstances.

Or either of the following may be used, according to the fancy of the doctor:

℞ Quina sulph ........................ grs. xxiv,
Cupri ........................ grs. xxxij,
Acidi sulphurici arom ........................ ℥ j,
Aqua pura ........................ ʒ xvj.

Fiat mistura et signa. Tablespoonful four times a day as a gargle. Or,

℞ Infus cinchon ........................ ʒ viij,

Mellis desp ........................ ʒ ij,

Acidi muriatici ........................ gts. xij.

Fiat gar gargarysma. This may be repeated several times daily."

**Resorcine in the Treatment of Purulent Vaginitis.**—The recent introduction of resorcine into therapeutics has developed some properties which render it especially applicable for external use. Cheron has employed it with success in the treatment of vaginitis purulenta, in both the acute and chronic stage. When there is much tenderness, so that a speculum cannot be introduced, a soft catheter or tube is pushed in, and irrigations of from six to ten minutes' duration are practiced three times a day of the following:

℞ Resorcin ........................ 10,

Aquæ fortis ........................ 1000.

M. As a result, the purulent discharge is rapidly reduced and the soreness subsides, so that a modification of the treatment may be made. He then applies,

℞ Resorcin ........................ 6,
Amyli glycerit ........................ 60.

M. This is to be carried to the bottom of the vagina, with the aid of the speculum, upon a tampon of cotton-wool, which is allowed to remain in place for from twelve hours to fifteen hours. The dressing is repeated every second day. Cure is thus obtained more rapidly than with the ordinary emollients and astringents.—*Le Progres Medical; Revue Med.-Chir. des Maladies des Femmes.*

**Eczema Rubrum of the Face aud Scalp.**—An infant aged seven months presents a marked form of this disease upon the cheeks and vertex, consisting of patches of inflammation, here and there oozing and covered with scales and adherent crusts. The case has been under treatment here for several days, and the lesions on the face are showing signs of improvement, while those on the scalp exhibit evidences of neglect, the crusts being allowed to remain. It is remarkable often how mothers, and even physicians, will object to the removal of these crusts. But as long as such effete material is permitted to remain, no remedies can be applied with success; it must, therefore, first be removed. After this has been done in this case, the following ointment will be ordered:

℞ Acid. salicylic.............................grs. xv,

Pulv. zinci oxid., } aa.........................ʒ j,

Pulv. amyli, }

Cosmoline..................................ʒ vj.

Sig. Apply twice a day.—*Med. and Surg. Rep.*

**On the Treatment of Whooping Cough.**—

℞ Croton-chloral..............................ʒ j,

Tr. cardam..................................℥ ij,

Tr. belladon.................................ʒ ij,

Glycerin....................................℥ iij.

M. Dose, half-teaspoonful to a child two years old every four hours. I have sometimes combined the several bromides with the croton-chloral, but I never felt sure that they added in any degree to its efficacy. If one bromide was better than another, it was the bromide of quinia. But I rely now exclusively on the croton-chloral in the management of pertussis. While I have never seen any unpleasant effects from this drug, I scarcely need add that in its exhibition a watchful care should be exercised lest, for some reason, its toxic effects should manifest themselves.—*Dr. W. C. Webb in Amer. Prac.—Med. and Surg. Rep.*

**Bryonia.**—Just one year ago. a gentleman 71 years of age came to see me, seeking relief for his swelled and paining hands and fingers (rheumatic, not gout). I gave him tincture of bryonia alba, a half teaspoonful to four ounces of water, one teaspoonful every two to four hours. He took it, and was relieved in a few days, regaining perfectly the functions of the members. To-day the man called again, having traveled about one hundred miles, to inform me that his skin disease was entirely cured, and wishing to know whether the medicine he took had also cured him of that malady, as he noticed an improvement from the beginning of taking the bryonia. In all he took one fluid ounce, when the cure was complete. The dermal perversion was ptyriasis of some 25 or 30 years standing, affecting the entire surface of the body. The furfuraceous desquamation was indeed terrible in the latter years; he would shed about one pint of this branny material in the course of a night. He had exhausted science and medicine during the time he was suffering, but without the least benefit.—*Cor. Ec. Med. Journal.*

